# Multi-pathway blood biomarkers to target and monitor multidimensional prevention of cognitive and functional decline (nested in the IN-TeMPO study framed within the world-wide FINGERS network)

**DOI:** 10.3389/fnagi.2025.1581892

**Published:** 2025-05-07

**Authors:** Gessica Sala, Luca Cuffaro, Federico Emanuele Pozzi, Simona Andreoni, Chiara Bazzini, Elisa Conti, Chiara Paola Zoia, Simone Beretta, Lucio Tremolizzo, Giuseppe Bellelli, Chukwuma Okoye, Maria Cristina Ferrara, Annamaria De Luca, Roberta Lenti, Paola Mantuano, Paola Pontrelli, Alessandra Stasi, Giovanni Defazio, Vincenzo Solfrizzi, Lucilla Crudele, Cristina Airoldi, Ferdinando Chiaradonna, Maria Pia Longhese, Giovanni Messina, Antonino Natalello, Ivan Orlandi, Alessandra Aloisi, Simonetta Capone, Assunta Ingannato, Benedetta Nacmias, Daniela Capello, Francesca Mangialasche, Carlo Ferrarese

**Affiliations:** ^1^School of Medicine and Surgery, University of Milano-Bicocca, Monza, Italy; ^2^Milan Center for Neuroscience (NeuroMI), University of Milano-Bicocca, Milan, Italy; ^3^Department of Neurology, Fondazione IRCCS “San Gerardo dei Tintori”, Monza, Italy; ^4^Acute Geriatric Unit, IRCCS “San Gerardo dei Tintori”, Monza, Italy; ^5^Unit of Pharmacology, Department of Pharmacy-Drug Sciences, University of Bari “Aldo Moro”, Bari, Italy; ^6^Department of Precision and Regenerative Medicine and Ionian Area (DiMePRe-J), University of Bari “Aldo Moro”, Bari, Italy; ^7^Department of Translational Biomedicine and Neuroscience (DiBraiN), University of Bari “Aldo Moro”, Bari, Italy; ^8^Department of Interdisciplinary Medicine, University of Bari “Aldo Moro”, Bari, Italy; ^9^Department of Biotechnology and Biosciences, University of Milano-Bicocca, Milan, Italy; ^10^Institute for Microelectronics and Microsystems, National Council of Research (CNR-IMM), Lecce, Italy; ^11^Department of Neuroscience, Psychology, Drug Research and Child Health, University of Florence, Florence, Italy; ^12^IRCCS Fondazione Don Carlo Gnocchi, Florence, Italy; ^13^Department of Translational Medicine, Centre of Excellence in Aging Sciences, University of Piemonte Orientale, Novara, Italy; ^14^Division of Clinical Geriatrics, Alzheimer Research Center, Department of Neurobiology, Care Sciences and Society, Karolinska Institute, Solna, Sweden; ^15^Theme Inflammation and Aging, Medical Unit Aging, Karolinska University Hospital, Stockholm, Sweden; ^16^FINGERS Brain Health Institute, Stockholm, Sweden

**Keywords:** blood biomarkers, aging, cognitive decline, senescence, prevention

## Abstract

**Background:**

As the population ages, the identification of preventive strategies able to delay cognitive and functional decline associated with aging represents a major challenge. To date, multidimensional approaches seem to be effective in reducing or delaying the onset of age-related diseases.

**Objectives:**

The multicentric randomized controlled trial IN-TeMPO (ItaliaN study with Tailored Multidomain interventions to Prevent functional and cognitive decline in community-dwelling Older adults, ClinicalTrials.gov ID NCT06248723), framed within the World-Wide FINGERS network, aims to verify the efficacy of guided multidomain interventions in preventing age-related cognitive and functional decline. Within this study, we will explore a comprehensive array of established and exploratory blood biomarkers of several pathologic age-related processes, including Alzheimer’s disease (AD), neurodegeneration, inflammation, senescence and sarcopenia, to stratify subject risk and assess the effect of multidomain interventions on biomarkers.

**Design and participants:**

ApoE4 status and plasma p-tau217 (AD), NfL (neurodegeneration), GFAP and IL-6 (inflammation), GDF-15 (senescence/sarcopenia) will be evaluated in all subjects (*n* = 1,662) both at the baseline and at the end of the study (12 months). Exploratory additional biomarkers will be measured at the same time points in a subgroup of 100 subjects: BDNF, ghrelin, IGF-1, irisin and redox status in plasma as markers of sarcopenia/senescence and oxidative stress, gamma-H2AX in PBMCs as marker of senescence, and amyloid beta aggregates in plasma, urine and erythrocytes as supportive markers of AD. Untargeted metabolomics analysis in plasma and untargeted volatilomics analysis in whole blood and urine will be performed to explore molecular alterations that may be associated with the pathogenesis and progression of age-related diseases in frail older adults with the aim of identifying novel potential biomarkers.

**Conclusion:**

The comprehensive clinical use of multiple laboratory biomarkers can contribute both to the early identification of trajectories of cognitive and functional decline in older adults, and to the identification of mechanisms underlying the effect of multidisciplinary interventions on age-related pathological processes.

## Introduction

### Background and aim

The aging of the population is a trend, unprecedented in human history, which affects the whole world, and Europe and Italy in particular. It has a pervasive impact on all sectors of society, including health and economy, with serious repercussions on the wellbeing of individuals, families and communities. The identification of preventive strategies to delay cognitive and functional decline associated with aging represents a major challenge that will provide healthcare systems with useful tools for the management of age-related disorders and disabilities (Livingston et al., 2024).

To date, multidimensional approaches seem to effectively reduce the social and economic burden of age-related diseases, improving the quality of life of individuals, their relatives, and societies. These approaches typically include a healthy diet, physical activity, cognitive stimulation, social activities, and the monitoring of risk factors related to cardiovascular and metabolic disorders. The Finnish Geriatric Intervention Study to Prevent Cognitive Impairment and Disability (FINGER) was the first trial to demonstrate that simultaneous lifestyle measures in five areas can improve brain health and prevent cognitive decline (Ngandu et al., 2015).

Following this seminal study, a growing number of clinical trials have been designed to further verify the feasibility and effects of multidomain interventions in preventing dementia and disability in elderly populations across various countries and settings. Currently, over 60 countries are part of this effort, coordinated within the World-Wide FINGERS (WW-FINGERS) global network for risk reduction and prevention of dementia (Kivipelto et al., 2020). These studies not only consider cognition, but focus also on the frailty syndrome, a state of increased vulnerability resulting from aging-associated decline in reserve and function.

Within the WW-FINGERS network, our group is currently conducting the multicentric ItaliaN Study with Tailored Multidomain Interventions to Prevent Functional and Cognitive Decline in community-dwelling Older adults (IN-TeMPO), a randomized controlled trial (RCT) in older people at increased risk of dementia and with mild to moderate frailty (ClinicalTrials.gov ID NCT06248723).

Only a limited number of similar studies include a comprehensive assessment of blood biomarkers to evaluate several pathologic processes which can help to predict intervention benefits and underscore their biological underpinnings (Ha and Son, 2018; Boardley et al., 2007). To address this gap, we resolved to leverage the IN-TeMPO trial to test the effect of multidomain interventions on a panel of established and exploratory blood biomarkers of Alzheimer’s disease (AD), neurodegeneration, inflammation, senescence, sarcopenia, and oxidative stress to stratify subjects´ risk and identify possible surrogate measures of the effect of multidomain interventions.

### Study population

The recruiting phase is currently ongoing within the IN-TeMPO study (ClinicalTrials.gov ID NCT06248723, protocol article in preparation), a RCT harmonized with the WW-FINGERS network, involving 9 clinical Italian centers (2 in the North, 2 in the Center, and 5 in the South). Briefly, a total of 1,662 community-dwelling older adults at increased risk of dementia and with mild to moderate frailty will be enrolled, according to the following inclusion criteria: age ≥ 60 years, Primary Care Frailty Index score between 0.07 and 0.21, Cardiovascular Risk Factors, Aging, and Dementia Risk Score (CAIDE) ≥ 6, Clinical Dementia Rating scale (CDR) ≤ 0.5, presence of an increased risk of developing dementia due to family history and/or at least one modifiable risk factor. The recruited subjects will be randomized into two groups. The first group will receive multicomponent interventions focusing on nutrition, physical exercise, cognitive training, management of metabolic and vascular risk factors, social activities, oral health, and recommendations for quality sleep. The second group will engage in self-guided interventions and will receive suggestions through app-web or video tutorials on the same domains. Wearable devices will be assigned to enrolled subjects to obtain clinical parameters. Informatic resources and artificial intelligence tools will be adopted to collect, analyze and share data. The primary objective is to evaluate the 12-month effect of multidimensional interventions on cognitive performance through a battery of neuropsychological tests (mNTB) in the active vs. the self-guided intervention group. Secondary and explorative objectives include assessing the impact of multidimensional interventions on functional and physical performance, as well as on a panel of blood biomarkers of cognitive and functional decline.

### Selection of blood biomarkers

**Figure 1:**
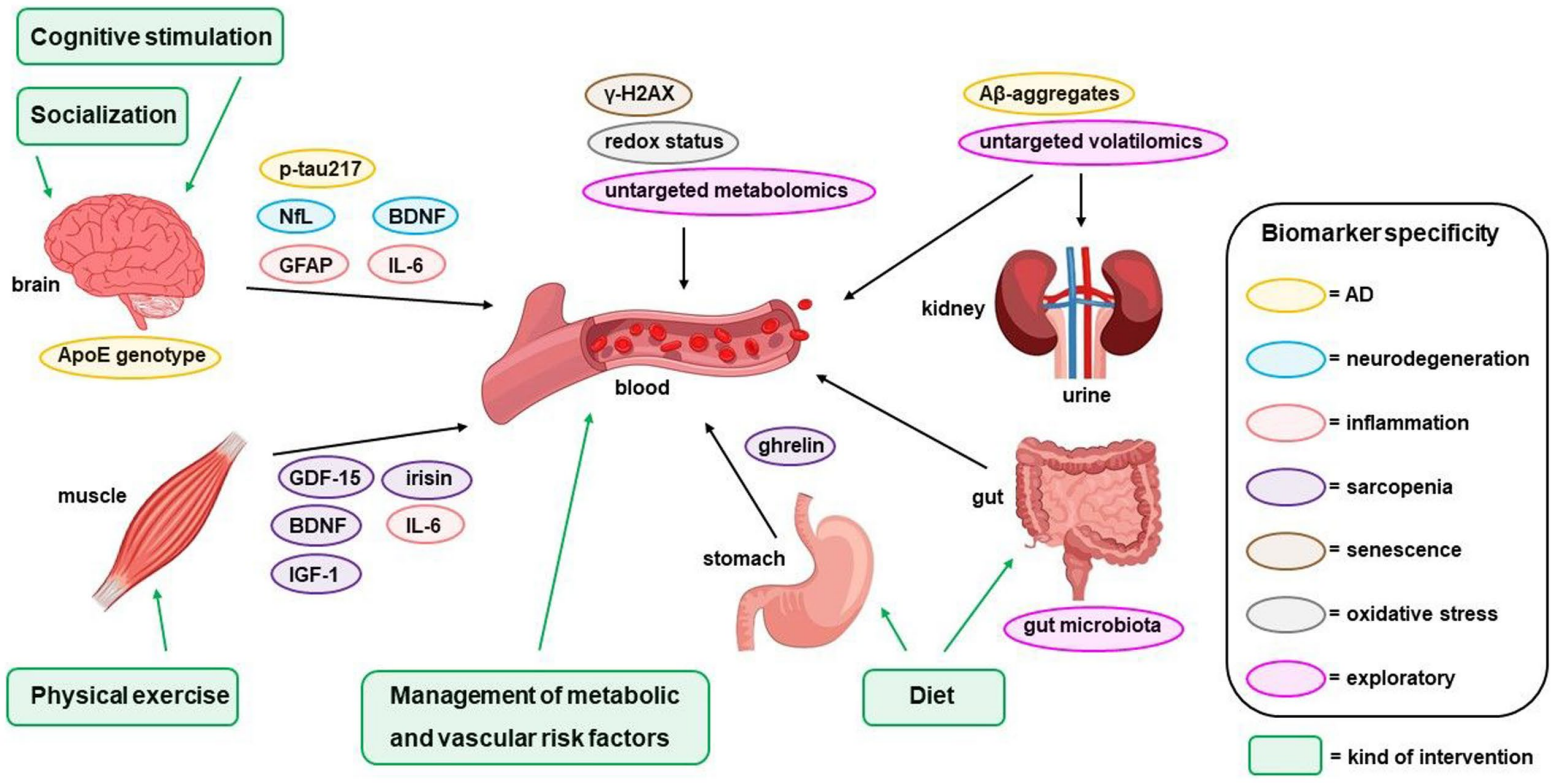
Blood biomarkers and multidomain interventions of the IN-TeMPO study.

A panel of blood biomarkers related to the main pathophysiological mechanisms underlying cognitive decline and physical frailty associated with aging will be analyzed in all subjects recruited in the IN-TeMPO study, both at the baseline and at the end of the study (12 months) (Table 1). The strategy that guided the selection of biomarkers was based primarily on expert consensus among the research team and the latest evidence from the literature on biomarkers related to In-TeMPO key outcomes [dementia and frailty (Salvioli et al., 2023)]. This selection was also driven by an adjunctive research hypothesis aimed at monitoring the pathways expected to be positively affected by the multidomain interventions. A schematic representation of all analyzed blood biomarkers and multidomain interventions planned for the IN-TeMPO study is shown in Figure 1.

**Table 1:** List of biomarkers evaluated in biological samples obtained from subjects participating in the IN-TeMPO study.

Biomarker	Specificity	Evaluated in:	Time pionts (months)	Biological sample	Sample storage temperature (°C)	Method
ApoE genotype	AD	All subjects	T0	Whole blood	-20	Real-Time PCR
p-tau217	AD	All subjects	T0, T12	Plasma	−80	CLEIA Lumipulse®
NfL	Neurodegeneration	All subjects	T0, T12	Plasma	−80	CLEIA Lumipulse®/ Simoa®
GFAP	Inflammation	All subjects	T0, T12	Plasma	−80	CLEIA Lumipulse®/ Simoa®
IL-6	Inflammation	All subjects	T0, T12	Plasma	−80	ELISA
GDF-15	Sarcopenia/senescence	All subjects	T0, T12	Plasma	−80	ELISA
γ-H2AX	Senescence	Subgroup (*n* = 100 subjects)	T0, T12	PBMCs	−80	Confocal microscopy
Redox status (NAD+/NADH, NADP+/NADPH, oxidized and reduced glutathione)	Oxidative stress/senescence	Subgroup (*n* = 100 subjects)	T0, T12	Plasma	−80	ELISA
Untargeted fingerprint	Senescence	Subgroup (*n* = 100 subjects)	T0, T12	Plasma	−80	FTIR spectroscopy
Metabolic fingerprint and profiling	Senescence	Subgroup (*n* = 100 subjects)	T0, T12	Plasma	−80	NMR spectroscopy
BDNF	Sarcopenia	Subgroup (*n* = 100 subjects)	T0, T12	Plasma	−80	ELISA
Ghrelin	Senescence/sarcopenia	Subgroup (*n* = 100 subjects)	T0, T12	Plasma	-80	ELISA
IGF-1	Senescence	Subgroup (*n* = 100 subjects)	T0, T12	Plasma	-80	ELISA
Irisin	Sarcopenia	Subgroup (*n* = 100 subjects)	T0, T12	Plasma	-80	ELISA
Untargeted volatilomics	Senescence/neurodegeneration/AD/inflammation	Subgroup (*n* = 100 subjects)	T0, T12	Whole blood, urine	−20	SPME-GC/MS, electronic nose
Protein aggregates	AD	Subgroup (*n* = 100 subjects)	T0, T12	Plasma, urine erythrocytes	−20 -80	AFM, spectrofluorimetry

The table lists all evaluated biomarkers, their specificity, the number of subjects analyzed, the time points, the type of biological sample and its storage temperature, and the method used. CLEIA, ChemiLuminescence Enzyme ImmunoAssay; Simoa, Single-molecule array; FTIR spectroscopy, Fourier-Transform Infrared Spectroscopy; NMR spectroscopy, Nuclear Magnetic Resonance spectroscopy; SPME-GC/MS, Solid-Phase MicroExtraction and Gas Chromatography/Mass Spectrometry; AFM, Atomic Force Microscopy.

#### Genetic risk factor

Apolipoprotein E (ApoE) genotype will be assessed in all recruited subjects at the baseline. ApoE ε4 is the most prevalent genetic risk factor for AD, increasing the likelihood of developing the disease by 2–3 times. New additional functions of ApoE on multiple amyloid beta (Aβ) -related or-independent pathways have been recognized (Raulin et al., 2022), opening the possibility of designing new effective ApoE-targeted therapeutic strategies for AD. Furthermore, multidomain interventions may be beneficial for cognition in older at-risk individuals even carrying the ApoE ε4 allele (Solomon et al., 2018; Sakurai et al., 2024; Andrieu et al., 2017; Hafdi et al., 2021) and recent studies are currently investigating whether such benefits are more pronounced in ApoE ε4 carriers compared with noncarriers (Forcano et al., 2021; Barbera et al., 2024).

#### Alzheimer’s disease continuum

Plasma phosphorylated-tau217 (p-tau217) has been selected as core blood biomarker of AD, based on its optimal performance to predict brain amyloid positivity found in the more recent literature (Ashton et al., 2023; Jack et al., 2024). Among the different p-tau isoforms, plasma p-tau217 displays the highest performance in both the early detection of AD pathology as shown by cerebrospinal fluid (CSF), showing the highest fold increase compared to the isoforms 181 and 231, and in differentiating AD from other neurodegenerative diseases in cognitively unimpaired subjects (Janelidze et al., 2023; Ashton et al., 2024). Increased p-tau217 levels are also associated with worsening brain atrophy and cognitive performance in AD patients (Ashton et al., 2022; Jonaitis et al., 2023). Furthermore, plasma p-tau217 assessment has recently been shown to predict the development of cognitive impairment up to 10 years later (Yakoub et al., 2024).

Aβ aggregates in plasma and erythrocytes will be analyzed as supportive markers of AD continuum in a subgroup of enrolled subjects (*n* = 100). Pathological protein aggregation is considered a key hallmark of a number of neurodegenerative diseases (Wilson et al., 2023). A previous study (Lan et al., 2015) and a more recent one (Nirmalraj et al., 2021) have yielded valuable preliminary insights into the clinical utility of nanoscale data gleaned from protein aggregate profiling on red blood cells (RBCs) of individuals experiencing memory issues and cognitive impairments. Through Atomic Force Microscopy (AFM) and the Thioflavin T (ThT) assay we aim to investigate the prevalence and the physical variations of AD associated-protein aggregates free in plasma or adsorbed on RBC membranes which may offer significant insights, at a systemic level, on the disease stage and progression. Moreover, as Aβ in circulating plasma is to some degree filtered into the urine of AD patients (Takata et al., 2008) we plan to implement an Aβ42 seed amplification assay (Salvadores et al., 2014) into urine samples of the same enrolled patients aiming to amplify and detect small amounts of misfolded Aβ oligomers glomerulus filtered.

#### Neurodegeneration

Neurofilament light chain (NfL), an intermediate filament with structural functions in the neuronal cytoskeleton, is a well-established biomarker of neurodegeneration. NfL levels greatly increase in both CSF and blood after axonal damage in many neurodegenerative conditions, including AD (Zetterberg et al., 2016). Albeit lacking specificity, NfL correlates with AD progression and is reported in the latest AD criteria as a biomarker of neurodegeneration (Jack et al., 2024). Interestingly, NfL levels seem to discriminate between different preclinical AD stages, such as Subjective Cognitive Decline (SCD) and Mild Cognitive Impairment (MCI) (Giacomucci et al., 2022), correlating with the disease progression; for this reason, it will be measured in all recruited subjects.

#### Neuroinflammation

Glial fibrillary acidic protein (GFAP) and interleukin-6 (IL-6) plasma levels have been chosen as inflammatory markers in the aging process.

GFAP is a class-III intermediate filament present mainly in astrocytes and involved in CNS architecture maintenance, cell communication and blood–brain barrier functioning. GFAP reflects early astrogliosis and neuroinflammation in AD, and recent evidence suggests that GFAP may increase before other well-known biomarkers (Fontana et al., 2023). Plasma GFAP levels are able to predict the risk of dementia in individuals with SCD and MCI (Ingannato et al., 2024; Wang et al., 2024), with an even better performance in plasma than in CSF (Wojdała et al., 2023; Benedet et al., 2021). However, similarly to NfL, the diagnostic value of plasma GFAP levels is strongly limited by non-specificity (Teunissen et al., 2022; Pichet Binette et al., 2023).

The pro-inflammatory cytokine IL-6, whose concentration increases in elderly subjects, is widely used as a sensor of inflammaging (Franceschi and Campisi, 2014). Together with TNF-*α*, IL-6 induces the production of C-reactive protein, another well-known age-associated inflammatory marker. Increased IL-6 levels are associated with geriatric syndromes including frailty, sarcopenia, and impaired functional capacity (Darvin et al., 2014).

#### Sarcopenia and muscle exercise

Growth/differentiation factor 15 (GDF-15) is a member of the TGF-*β* superfamily that is highly expressed in muscle tissue and will be used as a biomarker of senescence. Its expression is low in healthy, young subjects, with a range of 200–1,200 pg./mL (Breit et al., 2011). However, there is a notable increase in cases of chronic or acute disease conditions that may be age-related (Conte et al., 2019; Conte et al., 2020b). Furthermore, it is associated with a range of other conditions, including muscle atrophy, neurodegeneration, mitochondrial disease, and aging itself, which is independent of an individual’s health status (Lee et al., 2022; Liu et al., 2021). Moreover, GDF-15 has been proposed as a biomarker for sarcopenia, a clinical condition characterized by muscle atrophy and weakness (Kim et al., 2020; Herpich et al., 2021). Indeed, several studies have demonstrated a positive correlation between elevated serum levels of GDF-15 and a decline in skeletal muscle quality (Semba et al., 2020). While mounting evidence suggests a link between GDF-15 and sarcopenia, the precise function of GDF-15 in the reduction of muscle mass and function remains uncertain.

As additional biomarkers of sarcopenia and senescence, in a convenience sample of 100 enrolled subjects we will assess brain-derived neurotrophic factor (BDNF), a neurotrophin that plays a role in maintaining brain function. The concentration of BDNF varies throughout the lifespan, with generally higher levels observed during childhood than during adulthood. However, BDNF levels tend to be higher in early adulthood, with a gradual decrease observed over time (Webster et al., 2006). BDNF has also been associated with the processes of muscle repair, regeneration, and differentiation (Kalinkovich and Livshits, 2015). Importantly, BDNF plasma levels significantly increase with physical exercise, further supporting the role of exercise therapy in the prevention of cognitive decline (Coelho et al., 2014; Tsai et al., 2018). Therefore, BDNF may serve as a potential surrogate marker of sarcopenia and muscle exercise.

Another biomarker that will be evaluated in the same subgroup is ghrelin, a gastric peptide with GH-secretagogue activity that promotes the activation of the GH/IGF-1 axis, stimulates appetite and food intake, and is involved in the regulation of energy balance (Pradhan et al., 2013). It is well documented that ghrelin levels decline with age (Serra-Prat et al., 2007; Rigamonti et al., 2002). Despite the ongoing investigation into the relationship between ghrelin levels and sarcopenia, further research is required to fully establish this association (Serra-Prat et al., 2015).

We will be measuring insulin-like growth factor (IGF-1), a circulating hormone with an anabolic effect on muscle, the secretion of which declines throughout life (Chew et al., 2019). Normally, low levels of IGF-1 are associated with impaired muscle function and strength, making it potentially involved in the sarcopenic process (Roubenoff et al., 2003).

We will also study irisin, a myokine that plays an important role in energy regulation through the molecular mechanism known as “browning,” the conversion of mature adipocytes to a brown phenotype. It also improves glucose tolerance and reduces insulin resistance (Liu et al., 2022). Irisin levels are positively correlated with muscle mass and strength and tend to increase with physical training in humans and animals, while decreasing with age and under conditions of muscle atrophy and sarcopenia (Chang et al., 2017; Park et al., 2019), thus serving as a marker of the effect of muscle exercise.

#### Oxidative stress

Oxidative stress, resulting from the accumulation of reactive oxygen species (ROS) due to an imbalance between the oxidant-antioxidant systems, and energy metabolism impairment strongly contribute to cell damages associated with aging. In particular, nicotinamide adenine dinucleotide (NAD) metabolism plays an important role, not only in regulating ROS generation and antioxidant defence against oxidative stress, but also in cellular energy production. Furthermore, an altered mitochondrial respiratory activity and low levels of NAD metabolites (i.e., NAD+/NADP+) are associated to functional alterations in sarcopenic muscle. Consequently, monitoring the levels of key nicotinamide cofactors and glutathione in its reduced and oxidized forms are valuable biomarkers to assess the effectiveness of redox metabolism anti-oxidant defences.

#### Senescence

Phosphorylation of the histone variant H2AX producing *γ*-H2AX is a well-established molecular marker of double-strand breaks (DSBs), the most deleterious DNA lesions used as index of cell senescence. Indeed, quantitation of γ-H2AX foci by immunofluorescence represents a highly sensitive and specific method for the detection of these DNA lesions. The potential of γ-H2AX to act as a molecular marker in aging is emerging, and this is an area that is expected to be intensely investigated (Shreeya et al., 2023).

#### Untargeted –omics analyses of exploratory biomarkers

In addition to the study of specific biomarkers, untargeted analyses will also be performed in a subgroup of *n* = 100 enrolled subjects, aimed at identifying new biomarkers and/or specific molecular signatures related to aging. The decision to limit-omics analyses to 100 subjects is primarily based on the explorative nature of this part of the study and on the awareness that this sample size can be more than adequate to obtain robust indications on new possible biomarkers and pathways associated with aging. Furthermore, these analyses are high demanding in terms of execution times and costs, and the preparation of the biological samples necessary for these analyses is time-consuming and requires dedicated laboratory personnel not available in all recruiting clinical centers. Finally, the management and analysis of the large amount of results obtained with-omics techniques cannot be sustained in this study protocol on a larger number of subjects.

Untargeted metabolomics analysis via NMR spectroscopy will be performed in plasma, to provide both the “metabolic profiling” and the “metabolic fingerprint” of samples (Ghini et al., 2023). The metabolomic profiling is the total analysis of all metabolites assigned and quantified in samples, enabling the detection of changes in the metabolite concentrations, identifying potential biomarkers or metabolic pathways related to specific pathological conditions (Airoldi et al., 2016; Ciaramelli et al., 2017). On the other hand, the metabolomic fingerprinting relies on the analysis of spectral data, without prior signal assignment or metabolite quantitation, providing a rapid sample classification by multivariate statistical approaches, to discriminate between samples from different biological groups (i.e., before *vs* after treatment). Fingerprinting can be equated to a super-biomarker with superior discriminating power to the best single metabolite (or a group of the best metabolites). The NMR analysis of plasma metabolites has recently proven to be effective for the identification of cognitive decline and dementia markers in aged individuals (Conde et al., 2024).

Untargeted analysis of plasma will be also performed by Fourier transform infrared (FTIR) spectroscopy, which provides a snapshot of the molecular composition and structure of the investigated samples, called “spectroscopic fingerprint.” In particular, in the FTIR spectra of complex systems there is a combined and overlapping response of biomolecules, which, on one hand, prevents the identification of individual molecules but, on the other hand, allows for the simultaneous fingerprinting of different components (proteins, lipids, nucleic acids, and metabolites) (Baker et al., 2016). The spectroscopic fingerprint of plasma is expected to reflect the individual’s physio-pathological state. The application of machine learning-based methods allows for the classification of individuals based on the spectroscopic pattern and the identification of the spectral bands responsible for the classification accuracy provides information about clinical analytes. This enables the use of the spectroscopic fingerprint also for clinical phenotypes where the molecular origin is unclear (Baker et al., 2016). The potential use of IR spectroscopy, coupled with machine learning approaches, for the identification of spectroscopic biomarkers as useful diagnostic/prognostic tools has been reported (Baker et al., 2016; Paraskevaidi et al., 2017; Ami et al., 2021; Eissa et al., 2024).

Moreover, untargeted volatilomics analysis will be carried out in whole blood and urine to identify and measure those Volatile Organic Compounds (VOCs), part of the so-called human “volatilome,” that can be proposed as VOC biomarker patterns for AD and neurodegenerative diseases (González-Domínguez et al., 2017; Enche Ady et al., 2017). Volatilomics is indeed an emerging branch of metabolomics that studies biological systems as a whole identifying volatile metabolites within it for early diagnosis and exploration of mechanisms underlying a disease (Oxner et al., 2023; Hu et al., 2022). The human volatilome profile reflects biochemical processes that occur at the onset of a disease, and it can be extracted in non-invasive or semi-invasive mode from a series of biological samples (breath, urine, blood, saliva, feces, etc.) (Drabińska et al., 2021). Volatilomics-based approach for AD and neurodegenerative diseases is offering a novel tool to investigate the underlying pathologies (Conte et al., 2020a; Jiao et al., 2023; Lau et al., 2017; Tisch et al., 2013; Ubeda et al., 2022; Kimball et al., 2016; Conte et al., 2021). In this study we will use the VOC analysis in whole blood and urine to verify the effects of multidomain interventions on the sample population. The challenge is to extend basic knowledge about altered biochemical and metabolic pathways related to AD and neurodegenerative diseases, hence joining protein marker evidence in a novel integrated vision of such diseases.

### Biobank resource

Biomarkers discovery requires a critical mass of cross-sectional and longitudinal samples, along with high-quality associated data, all collected according to rigorous scientific criteria (Hulsen et al., 2019; Pepe et al., 2015). Research biobanks, highly organized repositories dedicated to the collection, processing, storage, and management of biological samples and their associated data in adherence to strict quality standards, have been established to address this critical need (Barnes and Watson, 2020; Annaratone et al., 2021). They ensure the ethical collection and use of these resources by adhering to legal frameworks (such as GDPR) and principles like FAIR (Findability, Accessibility, Interoperability, and Reusability). By providing well-characterized biological materials and comprehensive datasets, biobanks facilitate collaboration and represent a cornerstone for advancing scientific research (Malsagova et al., 2020).

UPO Biobank^1^ serves as the institutional research biobank of the University of Piemonte Orientale (UPO), housed at the Applied Research Centre Ipazia in Novara and integrated with the Regional Research Infrastructure Center of Autoimmune and Allergic Diseases (CAAD). UPO Biobank supports a wide range of research, including disease-specific investigations and population-based studies. It currently manages a repository of over 10,000 samples—including plasma, serum, urine, and PBMCs—collected from more than 3,000 participants.

Given its expertise in sample handling and its role in supporting molecular epidemiological research, UPO Biobank can significantly contribute to the IN-TeMPO study assisting participating centers in the systematic collection, processing, and storage of blood and other biological samples required by the study and facilitating the longitudinal assessment of blood biomarkers.

## Methods and analysis

### Sample collection and storage

**Figure 2:**
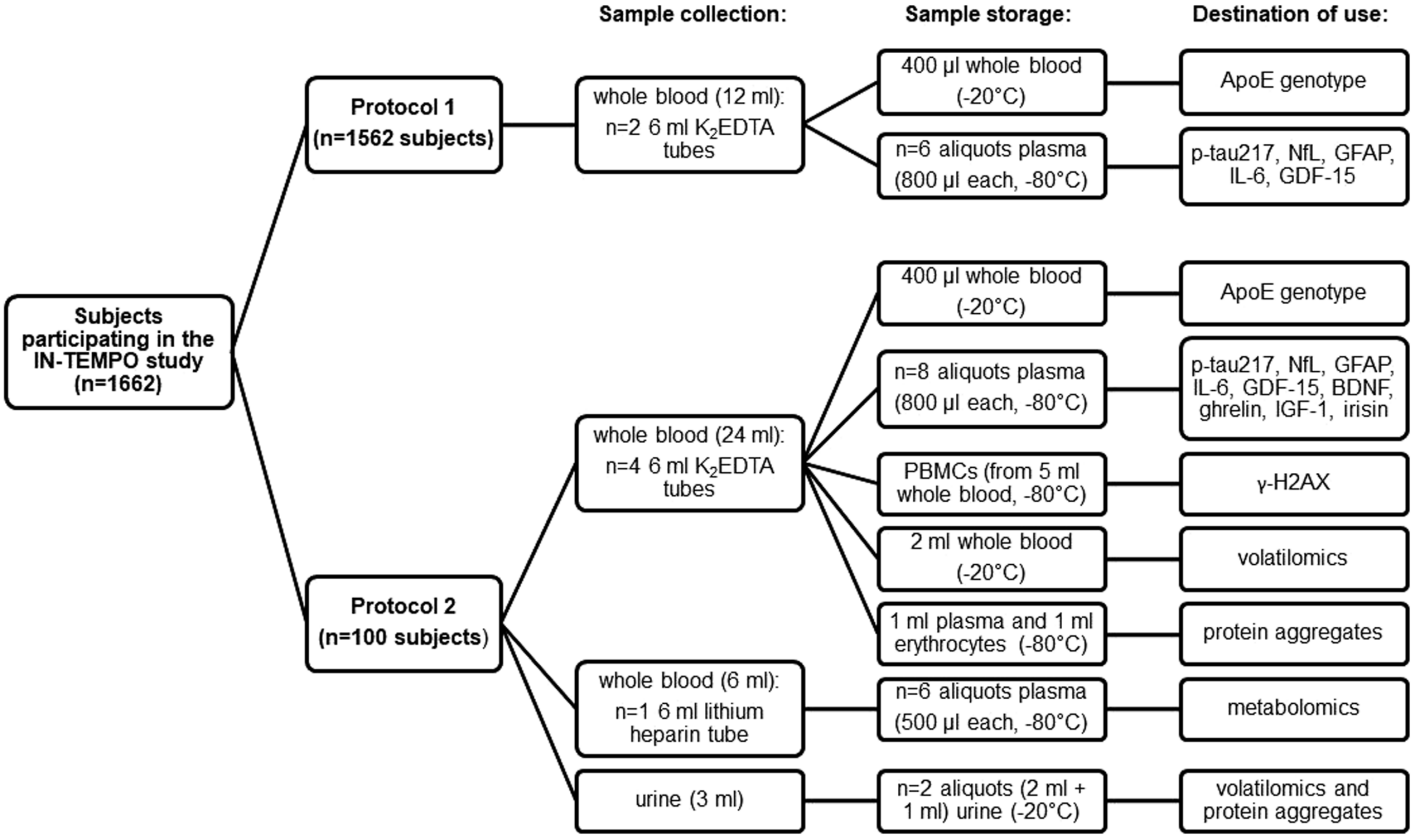
Schematic representation of the protocols for sample collection, storage, and destination of use.

As displayed in Figure 2, blood samples (12 mL) will be collected after overnight fasting in K_2_EDTA-containing tubes at the baseline and after the completion of active multidomain or self-guided interventions (12 months) from *n* = 1,662 subjects participating in the IN-TeMPO study, according to protocol 1. Protocol 1 will be applied in all clinical centers.

A 400 ul aliquot of whole blood will be frozen at−20°C for the assessment of ApoE genotype. The remaining blood will be immediately centrifuged (2000xg, 10 min, RT) to obtain plasma. Plasma will be aliquoted and frozen at−80°C until blind biomarker assessment or sample sending to laboratories responsible for carrying out the analyses.

For all collected samples, the analysis of p-tau217, NFL, GFAP plasma levels and of ApoE genotype will be performed in laboratories of the University of Milano-Bicocca and University of Florence, and that of IL-6 and GDF-15 in laboratories of the University of Bari and University of Piemonte Orientale.

In a subgroup of *n* = 100 subjects, recruited in the coordinating clinical center in Monza, protocol 2 will be adopted (Figure 2). According to protocol 2, blood samples (30 mL total, 24 mL in K_2_EDTA-containing tubes and 6 mL in lithium heparin tubes) will be collected after overnight fasting at the same time-points (baseline and after 12 months).

Besides the aliquots of whole blood and plasma described above, adjunctive whole blood (2 mL in headspace vials) and plasma aliquots will be prepared for the analysis of the additional plasma biomarkers (BDNF, ghrelin, IGF-1 and irisin), protein aggregation and volatilomic analyses.

For NMR-based metabolomics and FTIR-based molecular signature studies, plasma will be obtained as described above from whole blood with the anticoagulant lithium heparin, as requested by the specific methods (Beckonert et al., 2007).

Peripheral blood mononuclear cells (PBMCs) and erythrocytes will be prepared from 5 mL whole blood. More specifically, PBMCs will be isolated from whole blood by Lympholyte® (EuroClone) density gradient centrifugation. Briefly, blood samples will be diluted with the same amount of saline solution, layered on Lympholyte® and centrifuged (490 × g, 30 min, RT). PBMCs will be collected from the interface between plasma and Lympholyte®, washed with saline solution, and stored at−80°C in a cryoprotectant medium (RPMI 1640 + 10% fetal bovine serum) containing 10% dimethylsulfoxide (DMSO) until analysis of *γ*-H2AX. Erythrocyte pellets will also be collected, processed following glycerol-based cryopreservation (Rogers et al., 2018) and stored at−80°C for protein aggregate analysis.

Finally, 3 mL urine will be collected, aliquoted and stored at-20°C for volatilomic (2 mL in headspace vials) and protein aggregate analyses (1 mL).

The analysis of γ-H2AX and redox status, and the untargeted metabolic fingerprint and profiling will be performed in laboratories of the University of Milano-Bicocca. BDNF, irisin, IGF-1 and ghrelin plasma levels will be quantified in laboratories of the University of Bari. Untargeted volatilomics analyses in whole blood and urine and protein aggregates analyses in plasma/erythrocytses and urine will be done in laboratories of the CNR-IMM in Lecce.

### Biomarker analysis

ApoE genotype will be determined using DiaPlexQ™ Apolipoprotein E (ApoE) Genotyping Kit Real-Time PCR based assay (SolGent, Daejeon, Korea), after extraction of total DNA from whole blood with a commercial DNA extraction kit (Qiagen, Venlo, Netherlands).

Plasma p-tau217 levels will be measured with CLEIA (ChemiLuminescence Enzyme ImmunoAssay) using the Lumipulse® G600II instrument (Fujirebio), with a commercially available kit that includes specific quality controls.

NfL and GFAP levels will be measured with CLEIA (ChemiLuminescence Enzyme ImmunoAssay) using the Lumipulse® G600II instrument (Fujirebio) or the Simoa® (Single-molecule array) technology (Quanterix), instrument SR-X™, with commercially available kits that include specific quality controls.

IL-6, GDF-15, BDNF, ghrelin, IGF-1 and irisin plasma levels will be measured with commercially available ELISA kits (R&D Systems) via VICTOR Nivo™ multimode plate reader (Revvity, Waltham, MA, USA). Novel technologies, such as Simple Plex assay (Ella, ProteinSimple, Bio-Techne) for IL-6 and GDF-15, and high-throughput proteomics techniques (Olink platform) could also be used, according to manufactures instructions, for rapid and accurate processing of large number of samples or to gain more insight into identifying complex protein signatures, whenever needed.

Plasma NAD+/NADH, NADP+/NADPH and glutathione levels will be measured using microplate reader (Victor, ELx 800, Milano, Italy) with commercially available ELISA kits.

For γ-H2AX analysis, viable and untouched T cells will be purified from PBMCs by negative isolation (Dynabeads™ Untouched™ Human T Cells Kit, Thermo Fisher). Freshly isolated T cells will be cultured and expanded in a specific medium (CTS™ OpTmizer™ T Cell Expansion SFM, Thermo Fisher) in order to obtain frozen batches and for further analysis. Isolated T cells will be used to examine the phosphorylation status of histone H2AX, called γ-H2AX. Briefly, cells plated on 96-wells coated with fibronectin (FN), will be fixed with PFA and permeabilized with 0.1% (w/v) Triton X-100 solution diluted in PBS. γ-H2AX will be analyzed by using a H2A.X phospho S139 antibody-conjugated to Alexa Fluor® 488, followed by incubation with the nuclear stain 4,‘6-diamidino-2-phenylindole (DAPI). Fluorescence image analysis will be conducted on an Operetta CLS™ High-Content Analysis System (PerkinElmer, Waltham, MA, USA).

For the analysis of protein aggregates, aliquoted plasma and deglycerolized erythrocyte samples will be first investigated through thioflavin T (ThT) staining followed by immunofluorescence assay to confirm the presence of Aβ amyloid fibrils and/or aggregation. Twin samples of those revealing the ThT binding will be imaged by means of Nanosurf CoreAFM in air/tapping mode (after depositing ~50 μL or less of the sample on fresh cleaved mica substrate then allowing air drying). The size and morphology (ranging from spherical/annular oligomers, protofibrils/fibrils), the assembly patterns and prevalence of protein aggregates, from all conditions, will be quantified through analysis of height and phase-contrast AFM data.

On a parallel route, Cyclic Amplification of Aβ misfolding and aggregation will be performed exploiting the functional property of Aβ oligomers to seed the polymerization of monomeric Aβ. To this end, a commercial Aβ1-42 amyloidogenic peptide fragment will be used for *in vitro* aggregate production and aggregate free Aβ1-42 aliquots preparation. The experiment will be performed adapting to urine samples the protocol published by Salvadores et al. (2014), measuring ThT fluorescence at various time points reflecting the difference in the aggregation kinetic when it occurs.

For NMR metabolic analysis, frozen plasma samples will be slowly thawed at +4°C overnight and then mixed gently and centrifuged (3,400 × g, 3 min, +4°C) to remove possible precipitate. Aliquots of each sample will be transferred into 5-mm outer-diameter NMR tubes and mixed in 1:1 ratio with a phosphate buffer (75 mM Na_2_HPO4 in 80%/20% H_2_O/D_2_O, pH 7.4, including also 0.04% sodium azide). The NMR spectra will be acquired using a Bruker Avance III 600 MHz NMR spectrometer. The prepared plasma samples will be loaded onto a sample changer, maintaining the temperature of samples waiting to be measured at +20°C. Two NMR spectra will be recorded for each sample at 37°C. The first spectrum, an unedited presaturated proton spectrum, features resonances arising from all the sample components, including proteins and lipids within various lipoprotein particles. The second spectrum, a Carr-Purcell-Meiboom-Gill T_2_-relaxation-filtered spectrum, allows the suppression of the broad macromolecule and lipoprotein lipid signals, leading to enhanced detection of low-molecular-weight metabolites. Moreover, 2D total correlation spectroscopy (TOCSY) and heteronuclear single quantum coherence spectroscopy (HSQC) experiments will be acquired on a subset of samples to simplify resonance assignment (Beckonert et al., 2007; Julkunen et al., 2023). Metabolite identification and assignment will be performed with the support of 2D NMR experiments, the Human Metabolome Database, the Biological Magnetic Resonance Data Bank, and the SMA analysis tool^2^ integrated in MestreNova software.^3^ For metabolite quantification, the GSD (global spectrum deconvolution) algorithm, available in the MNova software package of Mestrelab will be exploited (Airoldi et al., 2016; Ciaramelli et al., 2017; Ciaramelli et al., 2018; Ciaramelli et al., 2023).

For the FTIR analyses, the frozen plasma sample will be thawed and centrifuged at 3400 × g, 3 min at +4°C. A few microliters of the sample will be deposited on the single-reflection diamond crystal of the system for measurements in attenuated total reflection (ATR, Quest, Specac). After the evaporation of the bulk water, absorption spectra will be collected by the Varian 670-IR spectrometer (Varian Australia Pty Ltd.) as previously described (Ami et al., 2019). The absorbance spectra and their second derivatives will be subjected to multivariate analysis as previously reported (Ami et al., 2021; Ami et al., 2019; Ami et al., 2024).

For volatilomic analysis, Volatile Organic Compounds (VOCs) from whole blood and urine samples collected in headspace vials will be extracted by headspace solid-phase microextraction (HS-SPME) according to an optimized protocol. Next, a Gas Chromatography/Mass Spectrometry (GC/MS) untargeted method for the analysis of VOCs profile of the biosamples, will be applied. The GC/MS analysis will be performed on a Shimadzu GCMS-QP2020X equipped with a split-splitless injector. A 624 gas chromatographic column (60 m x 1.40 μm x 0.25 mm) functionalized with a mid-polar stationary phase (6% Cyanopropyl-phenyl; 94% dimethyl polysiloxane) recommended for VOC analysis will be used. The identification of the volatile compounds is achieved by comparing mass spectra with those of the data system library (NIST 98, *p* > 80%). As a first approach, a semiquantitative method based on EPA 8260 Internal Standards Mix as internal standard will be used.

### Data analysis

Biomarkers value distribution and outliers will be checked. Logistic regression models will be used to predict AD using Lumipulse plasma p-tau217 > 0.14 pg./mL as a standard of truth, based on previously reported thresholds in Subjective Cognitive Decline (Contador J., Pozzi F.E., et al., Plasma and CSF biomarkers in Subjective Cognitive Decline: the BBRC Alzheimer at-risk (*Β*-AARC) cohort, personal communication at AD/PD™ 2025 Alzheimer’s & Parkinson’s Diseases Conference, April 1–5, Vienna). Biomarkers will first be tested individually as predictors (univariate models), and then in combination (multivariate models). Sensitivity, specificity, accuracy, and predictive values will be derived using Receiver Operating Characteristic (ROC) curve analysis. Optimal cut-offs will be determined based on the Youden index for univariate models. Differences in model performance (AUCs) will be assessed using the DeLong test. The influence of key demographic and genetic variables (age, sex, ApoE genotype) on biomarker distribution will be evaluated by including them as covariates in multivariate models. Adjusted and unadjusted models will be compared using Akaike (AIC) and Bayesian (BIC) Information Criteria.

Analyses of the main objective will include: (1) assess if baseline biomarkers levels can predict intervention response (e.g., heterogeneity of intervention effects) in order to identify biomarkers-based phenotypes/profiles with higher intervention benefits; (2) interventions effects on biomarkers levels; (3) relationship between change in biomarkers and change in clinical outcomes (cognition, frailty). Points 2 and 3 will help understand intervention effects on disease biomarkers and biological mechanisms of beneficial effects on different outcomes. Untargeted/exploratory-omics biomarkers will be analyzed with machine learning considering different predictors.

All *p*-values will be corrected for multiple comparisons using False Discovery Rate.

## Discussion

The identification of biomarkers represents an urgent need to standardize the methods of evaluating the aging-associated decline in physiological functions and the increased vulnerability to adverse outcomes characterizing the “frailty syndrome.”

Blood biomarkers have witnessed important development in the last decade due to both the availability of new ultrasensitive techniques for their detection in biological fluids, and the growth of –omics techniques. Importantly, our approaches can envisage, based on the experimental needs, the implementation of both classical and standardized ELISA assays with recently acquired advanced ultrasensitive methods, such as the Ella™ Automated ELISA that enables robust biomarker analysis in large-scale studies, as well as with high-throughput proteomics techniques like the Olink platform, which facilitates the identification of complex protein signatures of biological aging.

Recent studies have attempted to correlate some clinically relevant biomarkers with the risk of cognitive and physical decline in aging. Focusing on cognitive decline, the current model recognizes the existence of a progression from intact cognitive performance to dementia, which includes the phases of SCD and MCI. In each of these phases, it is possible to make use of specific biomarkers to identify *in vivo* the presence of AD and the extent of neurodegeneration, according to the ATN classification proposed by the NIA-AA (Jack et al., 2018) and the International Working Group (Dubois et al., 2014), and updated in the new criteria published last year (Jack et al., 2024). In AD, blood biomarkers are emerging as scalable tools for clinical evaluation, trial recruitment, and disease monitoring. An excellent diagnostic accuracy of plasma biomarkers of Aβ and tau obtained with ultrasensitive techniques (Pais et al., 2023; Sarto et al., 2023) has recently been demonstrated, and other potential blood biomarkers, including NfL, GFAP, BDNF are of particular interest in the early stages of cognitive decline (Giacomucci et al., 2022; Kim et al., 2023). Recently, a serum dysregulation of serine and glycine metabolism has been also described as a potential predictive biomarker for cognitive decline in frail elderly subjects (Imarisio et al., 2024).

When considering results from-omics techniques, as an example, a panel of 22 biomarkers associated with functional and cognitive decline and hypomobility has been identified through metabolomics analyses (Kameda et al., 2020).

Due to the high demand in terms of both organizational complexity and personnel and laboratory reagents costs, only a limited number of studies involving community-dwelling older adults included the analysis of the effect of lifestyle-based multidomain interventions on blood biomarkers associated with cognitive and functional decline (Ha and Son, 2018; Boardley et al., 2007), although some studies are currently ongoing. For this reason, the inclusion of a comprehensive array of blood biomarkers associated with the main mechanisms of aging represents a great added value to the IN-TeMPO study, and a unique opportunity to advance knowledge in this line of research.

## Limitations

Although we are aware that a combination of several blood biomarkers may be more accurate with respect to the single or a limited number of biomarkers, due to practical budget limitations, we were forced to choose what to date appears to be the best plasma biomarker candidates for mechanisms of our interest in the context of the IN-TeMPO study.

As an example, we choose to analyze plasma levels of p-tau217 as best candidate biomarker for AD, even if recent literature findings indicate that combining plasma beta-amyloid and p-tau217 may improve the detection of brain amyloid in non-demented elderly (Niimi et al., 2024). Similar motivations have led to the choice of a limited number of biomarkers, among all interesting ones, for other mechanisms related to aging, including inflammaging, senescence and sarcopenia. When considering the complex phenomenon of inflammaging, the assessment of IL-6, whose levels depends on multiple stimuli, seems to be not sufficient to verify the actual association with aging, and the evaluation of IL-6 soluble receptor (sIL-6R), a marker of the regulatory counterpart of inflammaging known as anti-inflammaging (Franceschi et al., 2007), should be added to increase the result consistency.

However, almost all biomarkers chosen for IN-TeMPO are measured also for FINGER study, and, given the fact that there is prospective data harmonization among the 2 RCTs, joint data analysis will increase statistical power and enable validation of findings in a more heterogeneous population (North and South of Europe).

Collectively, it would certainly be desirable to increase the number of biomarkers investigated to have a more comprehensive knowledge on the effect of multidimensional interventions on a wider panel of blood biomarkers in elderly subjects. Untargeted metabolomic analyses, although planned in a subgroup of recruited subjects, were envisaged in this study to partially overcome this issue. Unfortunately, the subgroup of 100 enrolled subjects cannot be representative from Northern, Central and Southern Italy, as only the coordinating clinical center in Monza (Northern Italy) has laboratory personnel dedicated to preparing samples for these analyses.

Moreover, we are currently exploring possible implementation of this study protocol with further grants to: (i) evaluate third generation epigenetic clocks, to compute the rate of biological aging (Belsky et al., 2022) and to predict cognitive decline progression (Sugden et al., 2022) with even better performance than blood-based biomarkers (Grodstein et al., 2021); (ii) analyze gut microbiota, whose composition in terms of abundance of specific microbial strains seems to correlate with age and to represent a risk factor for many age-related disorders; (iii) build a biobank including several additional biological samples (saliva, tears, RNA) for future high-throughput metabolomics and transcriptomics analyses.

## Conclusion

The clinical use of laboratory biomarkers can contribute to the early identification of trajectories of cognitive and functional decline, offering the possibility to anticipate the implementation of currently available preventive strategies against this age-related condition.

The analysis of clinically relevant biomarkers in accessible biological samples from participants will contribute to the understanding of the complex mechanisms underlying the effect of multidomain interventions on several pathological processes closely associated with age-related decline. Ultimately, this will drive precision prevention of age-related cognitive and functional decline, with the definition of multidomain interventions models for different risk profiles, maximizing therapeutic benefits.

## Footnotes

^1^ https://biobank.uniupo.it/

^2^ https://mestrelab.com/main-product/sma-mixtures-analysis

^3^ https://mestrelab.com/main-product/nmr
